# VNS-BA*: An Improved Bidirectional A* Path Planning Algorithm Based on Variable Neighborhood Search

**DOI:** 10.3390/s24216929

**Published:** 2024-10-29

**Authors:** Peng Li, Ying Li, Xuesong Dai

**Affiliations:** 1 School of Electronic and Information Engineering, Nanjing University of Information Science and Technology, Nanjing 210044, China; 2 School of Automation, Wuxi University, Wuxi 214105, China

**Keywords:** A* algorithm, path planning, mobile robot, bidirectional search, global planning

## Abstract

The A* algorithm is an effective method for path planning; however, it has certain drawbacks, such as a high number of turns, low planning efficiency, and redundant searches. To address these issues, this paper proposes an improved bidirectional A* global path planning algorithm based on a variable neighborhood search strategy, named VNS-BA*. The new algorithm first employs an 8-11-13 neighborhood search method for node expansion. Then, the bidirectional search strategy is optimized by using the current nodes of the opposite path and the global target point, enabling the two paths to meet in the middle of the map. Finally, redundant turns are removed from the path, and cubic spline interpolation is applied to achieve local smoothing at the turns. The effectiveness of the improved algorithm was validated on different maps and compared with A* and its three derived improved versions. The simulation results indicate that VNS-BA* shows significant improvements in terms of the number of path turns, turn angles, and planning efficiency.

## 1. Introduction

Path planning is a critical technology for mobile robots, essential for executing dynamic tasks efficiently and safely in specific scenarios. Path planning can be divided into two main categories based on prior environmental information: global path planning and local path planning. Global path planning constructs an optimized path from the starting point to the target point in a static environment, where information such as the position and distribution of obstacles is known in advance. In contrast, local path planning, while capable of refining the path online based on dynamic information about unknown obstacles, may still be prone to getting trapped in local minima due to the lack of global environmental information. As a result, global planning is a crucial preliminary step, with the quality of the global path directly impacting navigation performance.

To address global path planning, several graph-based search algorithms have been proposed, including Breadth-First Search (BFS) [1], Depth-First Search (DFS) [2], and Dijkstra’s algorithm [3]. These algorithms offer high path coverage but suffer from low efficiency due to redundant searches. Sampling-based algorithms, such as Rapidly-exploring Random Trees (RRT) [4], RRT-connect [5], Probabilistic Roadmaps (PRM) [6], and lazy PRM [7], provide unique advantages for real-time applications but often fail to fully capture environmental details, leading to suboptimal paths. In subsequent developments, bio-inspired algorithms like Ant Colony Optimization (ACO) [8], Particle Swarm Optimization (PSO) [9], and Whale Optimization [10], as well as machine learning methods based on reinforcement learning [11] and deep reinforcement learning [12,13,14], have also been widely applied to global path planning. While these algorithms are capable of generating high-quality paths and have relative advantages in handling complex and continuous search spaces, they generally require substantial resources, which makes them challenging to deploy in resource-constrained systems.

The A* algorithm is a global path planning method based on graph search, which improves upon blind search algorithms by utilizing heuristic information from the map to selectively expand path nodes. This provides advantages such as high search efficiency and simplicity. Due to its low computational resource requirements and minimal memory usage, A* is widely employed in practical robotic systems, such as Automated Guided Vehicles (AGVs) [15,16] and Unmanned Ground Vehicles (UGVs) [17]. However, traditional A* has limitations, including excessive path turns and redundant searches. Numerous scholars have worked to address these issues. Zhang et al. proposed Rectangular Expansion A* (REA*), which replaces the traditional eight-neighbor expansion of A* with a rectangular expansion, significantly enhancing its performance. Xu et al. further improved REA* by introducing bidirectional rectangular expansion, effectively reducing the number of expansions and accelerating the algorithm [18]. However, rectangular expansion significantly increases computation time in environments with high obstacle density. Liao et al. enhanced the heuristic function by adding adaptive weight factors to improve path safety and searching speed [19]. Kim and Ha used methods based on Artificial Neural Networks (ANN) and reinforcement learning to train the heuristic function, thereby continuously improving algorithm performance during the learning process [20,21]. While these approaches improved the heuristic function, there remains room for optimization regarding path turns. Bai and Xu utilized the Floyd smoothing algorithm to eliminate unnecessary path nodes [22,23]. These improvements have enhanced A* in specific aspects but are not comprehensive.

To address the issues of excessive node expansions, large turning angles, and unsmooth paths in the traditional A* algorithm, this paper proposes an integrated improvement called the VNS-BA* algorithm. The proposed algorithm employs a simultaneous bidirectional search strategy from both the start and goal points, combined with a variable neighborhood search strategy, an improved heuristic function, and path smoothing operations, significantly enhancing search efficiency and path quality. The validation results indicate that the algorithm performs well across various maps of differing sizes and complexities, providing a new path planning solution for mobile robots in environments such as indoor settings, ports, and warehouses.

The specific improvements are as follows:

1. The traditional eight-neighbor search is expanded to an 8-11-13 neighbor search. Nodes are selectively expanded based on the positional relationship between the target point and the current node, reducing redundant searches.
2. An improved bidirectional search strategy is employed, where the search begins simultaneously from both the start and target points, using the current node from the opposite path as the target point.
3. The heuristic function is modified by introducing a new distance function that incorporates the current distance to the opposite path and a weighted distance to the goal. Additionally, the obstacle density between the current and target nodes is included as a weighting factor.
4. Pruning operations are performed to eliminate unnecessary turns in the path.
5. Cubic spline interpolation is applied to locally smooth the path at turn points.

## 2. A* Path Planning Algorithm

The traditional A* algorithm relies on a pre-established environmental model to plan a collision-free and safe path from the starting point to the destination. Currently, grid maps are widely used. In this approach, the map is divided into several independent square grids, each assigned a value based on the presence of obstacles. In this paper, we apply a simple binary treatment to the grids: grids containing obstacles are assigned a value of 1 and displayed in black on the map, while passable areas are assigned a value of 0 and displayed in white. To ensure that the generated path does not collide with obstacles, the grid size is set to the sum of the robot’s maximum diameter and the designated safety distance. This ensures that even if the path diagonally touches the corner of an obstacle grid, there will be no collision. After acquiring the map information, the A* algorithm selects nodes (i.e., grid centers) with the lowest path cost based on the cost function, and expands them until the destination is reached. The optimized path is the one with the minimum total cost, as determined by the algorithm.

The path cost function f(n) is the sum of the actual cost from the start point S to the current node n, denoted as g(n), and the estimated cost from the current node n to the goal G, denoted as h(n), as shown in Equation (1). The traditional A* algorithm typically uses Euclidean distance (Equation (2)), Manhattan distance (Equation (3)), and Chebyshev distance (Equation (4)) to express the estimated cost. In obstacle-free environments, Euclidean distance provides the most direct estimation of cost. However, when obstacles exist between two points, the robot must navigate around them, causing the path to include turns. In such cases, Manhattan distance can more accurately represent the estimated cost. In this paper, we switch the distance estimation function used for h(n) based on whether obstacles exist along the line connecting the current node and the target point.

(1) f(n)=g(n)+h(n)

(2) $$h{\left(n\right)}_{Euclidean}=\sqrt{{\left(x_{n}-x_{G}\right)}^{2}+{\left(y_{n}-y_{G}\right)}^{2}}$$

(3) $$h{\left(n\right)}_{Manhat\tan}=\left|x_{n}-x_{G}\right|+\left|y_{n}-y_{G}\right|$$

(4) $$h{\left(n\right)}_{Chebyshev}=\max(\left|x_{n}-x_{G}\right|,\left|y_{n}-y_{G}\right|)$$

The specific implementation of the A* algorithm is as follows:

1. Initialize two node lists: open list and close list.
2. Begin the search from the start point and add it to the open list.
3. Check whether the target point is in the close list. If true, trace back from the target point through the parent nodes to generate a complete path, and the pathfinding process is finished. If false, proceed to the next step.
4. Move the node with the smallest f(n) value from the open list to the close list, and set it as the current node.
5. Search the neighboring nodes of the current node and add all reachable, non-obstacle nodes to the open list.
6. If the expanded node is not in the open list, set the current node as its parent. If the expanded node is already in the open list, recalculate its g(n). If the updated g(n) is smaller, set the current node as its parent. If the updated g(n) is larger, retain its original parent node.
7. Repeat steps 3 to 6 until the loop terminates, indicating that the path search is complete.

## 3. Improved Bidirectional A* Algorithm

### 3.1. Adaptive 8-11-13 Variable Neighborhood Search

The traditional A* algorithm expands nodes around the current node, and common search strategies are typically divided into 4-neighborhood and 8-neighborhoo searches based on the expansion directions. The 4-neighborhood search only expands nodes in the four cardinal directions (up, down, left, and right) from the current node, restricting movement to horizontal and vertical directions. This leads to excessive path turns, and since the number of nodes explored per iteration is small, multiple iterations are required during the search process. The 8-neighborhood search adds four diagonal directions to the 4-neighborhood search, reducing the number of turns and shortening the overall path length compared to the right-angled paths of the 4-neighborhood search. The search process also requires fewer iterations, improving search efficiency. It performs well in most scenarios; however, because it expands uniformly in all directions, it results in redundant searches in directions that deviate from the target.

To address these issues, the proposed VNS-BA* algorithm employs an adaptive 8-11-13 variable neighborhood search strategy. This strategy expands the search neighborhood adaptively and switches between search strategies based on surrounding obstacles, selectively expanding nodes based on the target’s position.

**Figure 1:**
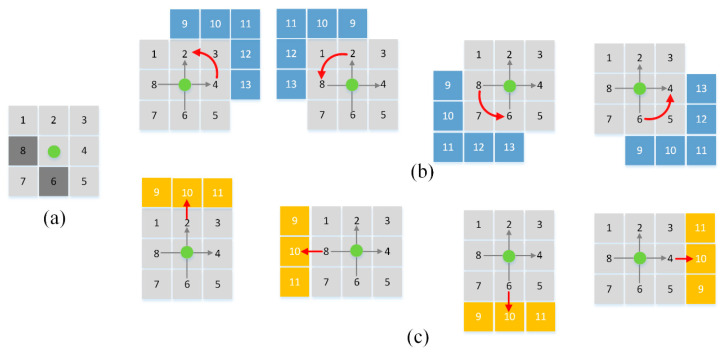
(**a**) 8-neighborhood search, (**b**) 13-neighborhood search, (**c**) 11-neighborhood search. The green node represents the current node, light gray grids indicate the 8-neighborhood search area, dark gray grids represent obstacle grids, blue grids indicate the additional grids expanded by the 13-neighborhood search, yellow grids represent the additional grids expanded by the 11-neighborhood search, and the red arrow shows the vector direction from the target point to the current node.

When expanding nodes, the algorithm first visits the nodes surrounding the current node using the 8-neighborhood search method. If no obstacles are present in the eight neighboring areas, the search continues outward. A vector from the current node to the target node is calculated, with the current node as the origin. If the vector aligns with the axes centered on the current node, three additional nodes are expanded in the vector’s direction, forming an 11-neighborhood search. If the vector lies within one of the four quadrants of the coordinate system, five additional nodes are expanded in the direction of the corresponding quadrant. However, if obstacles are present in the initial 8-neighborhood search, the outward expansion is halted, and only the 8-neighborhood search is performed, preventing the path from crossing obstacles and ensuring safety. As illustrated in Figure 1.

Zhang et al. [24] and Sun et al. [25] also proposed search strategies that expand the 8-neighborhood to a 24-neighborhood or 48-neighborhood, uniformly expanding in all directions. While these approaches improve search efficiency, they lack directionality. The method proposed in this paper adapts the search towards the target direction, significantly reducing redundant searches that deviate from the goal. Additionally, by increasing the number of nodes expanded per iteration, the number of traversals is reduced, which also decreases the number of turns in the path.

### 3.2. Improved Bidirectional Search Strategy

**Figure 2:**
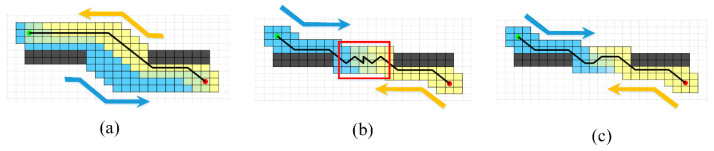
(**a**) General bidirectional search strategy, (**b**) the improved bidirectional search strategy proposed by Li et al. [26], (**c**) the bidirectional search strategy proposed in this paper. In the figure, the red node represents the start point, the green node represents the goal point; yellow grids indicate the grids traversed by the forward path search, and blue grids represent the grids traversed by the backward path search. The yellow arrow points in the direction of the forward path search, and the blue arrow points in the direction of the backward path search.

The traditional A* algorithm uses a unidirectional search from the start to the goal, which is inefficient in large maps with numerous obstacles. To address this limitation, scholars have integrated the bidirectional search strategy with the traditional A* algorithm, resulting in the Bidirectional A* (BA*) algorithm. In BA*, searches are initiated simultaneously from both the start and goal points, with each treating the other as the target, until the paths meet in the middle. Then, the complete path is generated by backtracking from the meeting point to the start and goal. In theory, this approach can reduce search time by approximately 50% compared to unidirectional searches. However, it has a significant drawback: the directions of the two paths may diverge, failing to meet in the middle of the map, leading to redundant searches. As a result, the total number of expanded nodes may even exceed that of a unidirectional A* search, consuming additional computational resources, as shown in Figure 2a.

Li et al. [26] proposed an improved bidirectional alternating search strategy, where the forward and backward paths each use the current best node of the opposite path as the target. This ensures that the two paths intersect in the middle to form a complete path. However, this method still has limitations. First, because the target node’s position is continuously updated, disturbances can occur when expanding nodes forward, leading to excessive turns in the generated path, or even a zigzagging pattern, as illustrated in Figure 2b. Additionally, due to the lack of global guidance from both the start and goal points, “detours” may occur. Furthermore, the final backtracking to generate the complete path only begins when the current nodes of both paths coincide, leading to unnecessary searches.

To address the aforementioned issues, VNS-BA* employs an improved bidirectional search strategy, implemented by modifying the heuristic function. In a typical bidirectional A* algorithm, the heuristic function is defined as the distance from the current node to the start of the opposite path (usually using Euclidean or Manhattan distance). In the proposed strategy, the heuristic is defined as the distance from the current node n to the current node on the opposite path h(n,oppsite_current) and the distance from the current node to the start of the opposite path h(n,oppsite_start), each assigned different weight coefficients, ω,η. The weighted sum of these distances is used as the distance function p(n), while the rectangular obstacle density between the current node and the opposite path’s current node is used to construct an obstacle weight factor $e^{\rho }$.

The product of the obstacle weight factor and the distance function is used as the heuristic function h(n), as shown in Equations (5) and (6). It is important to note that the distance function switches between Euclidean and Manhattan distances based on the obstacle conditions between two points, as described in Section 2. The actual cost g(n) is defined as the Euclidean distance between the current node and the start node, as shown in Equation (7).

(5) p(n)=ω×h(n,oppsite_current)+η×h(n,oppsite_start)

(6) $$h(n)=e^{\rho }\times p(n)$$

(7) $$g(n)=\sum_{i=1}^{n}\sqrt{{\left(x_{i-1}-x_{i}\right)}^{2}+{\left(y_{i}-y_{i-1}\right)}^{2}}$$

The function h(n,oppsite_current) allows the current path to move closer to the opposite path, ensuring that the two paths intersect in the middle of the map. The function h(n,oppsite_start) provides overall guidance, directing the current node towards the final target, which helps avoid repeated local searches and reduces the disturbances caused by frequent changes in the opposite path during dynamic planning. This approach enhances system robustness and minimizes turns along the path, as shown in Figure 2c.

**Figure 3:**
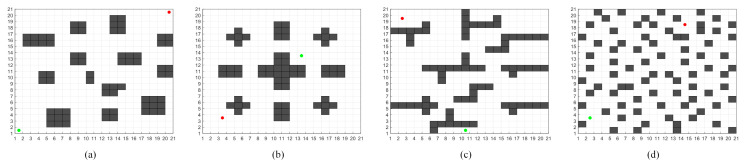
(**a**) Simple map, (**b**) Map with obstacles placed in the middle, (**c**) Map with bar-shaped obstructions, (**d**) Random map with 20% obstacle density. In the figure, the red node represents the start point, the green node represents the goal point.

The weight coefficients, ω and η, define the attraction of the current node n on the opposite path, oppsite_current, and the pull of the start node on the opposite path, oppsite_start, toward n. When ω is large, the current path aligns more with the opposite path, particularly when a significant obstacle exists between them. The current path may adjust to closer nodes around the obstacle rather than proceeding directly to the target node. This adjustment reduces search time but may increase the number of nodes explored and the overall path length. Conversely, when η is large, the current path focuses more on reaching the target, possibly encountering fewer obstacles, though with higher search costs and reduced efficiency. To determine appropriate values for the weight coefficients, ω and η were set between 0 and 1, with a step size of 0.1, as illustrated in Figure 3, which shows results from four test scenarios. As this study focuses on optimizing paths through offline experiments, the effects of turning angles and frequency were omitted, using instead the number of nodes explored and the search time as key metrics. The results in Table 1 show that ω’s effect on overall path length is minimal. However, when ω and η are set to 0.5, both the number of nodes explored and the search time are minimized. Thus, for the subsequent experiments and simulations, both ω,η are set to 0.5.

**Figure 4:**
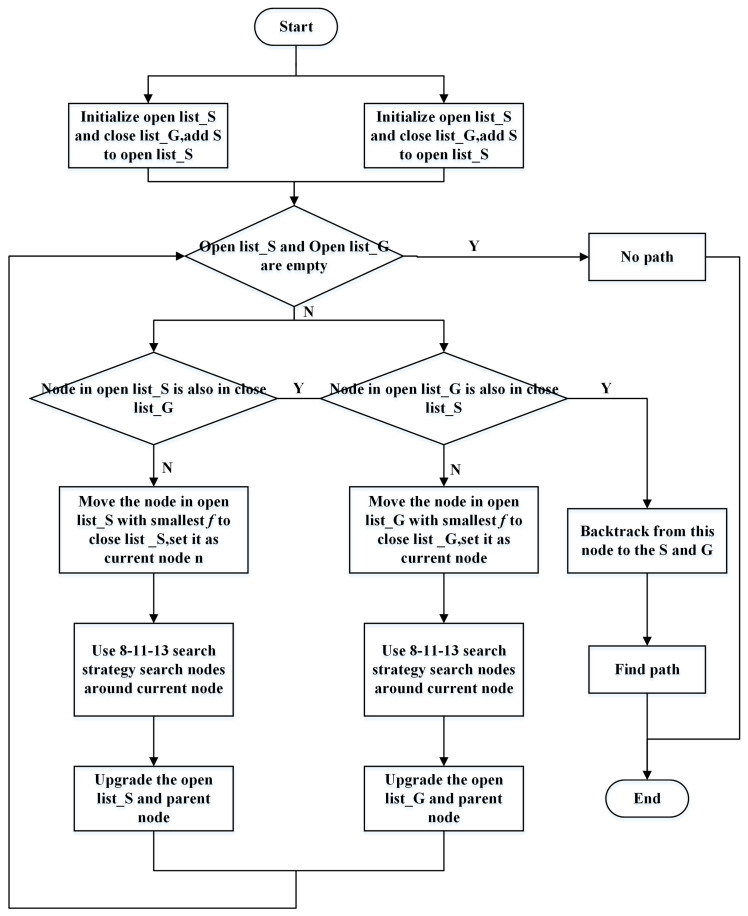
Flowchart of the improved bidirectional A* algorithm proposed in this paper.

Generally, as the number of obstacles increases, the probability of detours increases, resulting in longer path lengths. The obstacle weight factor $e^{\rho }$ helps ensure that the heuristic function h(n) better reflects the actual cost when the path crosses areas with uneven obstacle distribution and guides the path toward regions with fewer obstacles. The algorithm flowchart is shown in Figure 4:

### 3.3. Path Polyline Optimization

The initially planned path may contain unnecessary turns. This paper proposes a method to perform a secondary optimization on the initially generated path to remove redundant turning points, making the path more in line with the kinematic model of mobile robots.

**Figure 5:**
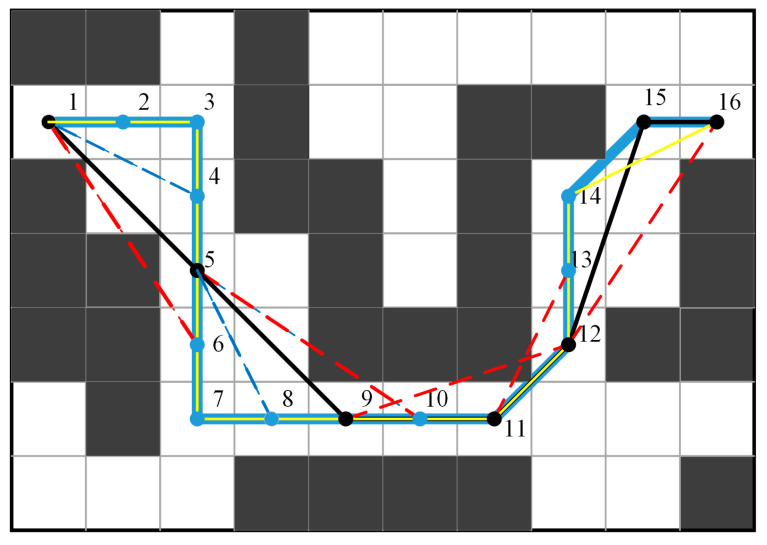
Illustration of polyline optimization. The blue line represents the initial path, the yellow line represents the path optimized by Huang et al. [27], and the black line represents the path optimized in this paper.

To optimize the initial path, Huang et al. [27] first removed collinear redundant nodes from the path and then eliminated unnecessary turning points. While this approach can optimize the path to some extent, particularly in terms of length, it does not completely remove redundant turns. When dealing with longer paths with more turns, the optimization effect is less pronounced. We simulated the implementation of this algorithm in the map environment shown in Figure 5. The blue line represents the initial path, and the blue nodes indicate the initial nodes. After removing collinear redundant nodes, the path nodes become 1-3-7-11-12-14-15-16. Then, after removing additional unnecessary turns, the path nodes become 1-3-7-11-12-14-16. The optimized path is shown by the yellow line. Compared to the original path, this method only reduces one turning point, indicating that there is still room for further optimization.

The specific idea of the algorithm proposed in this paper is as follows: First, the start point of the path is added to the optimized path list, and the start point is set as the front endpoint. Subsequently, the remaining nodes of the path are sequentially connected as the back endpoint to form a line segment. It is then determined whether the line segment crosses any obstacle grids, as indicated by the blue dashed line in the figure. The path nodes are traversed until a node forms a line segment with the start point that crosses an obstacle, as shown by the red dashed line in the figure. At this point, the previous node of the crossed node is added to the optimized path list, and this node is set as the new start point. The above steps are repeated, continuing to traverse the original path nodes until the end point is reached. The end point is then added to the optimized path list as well. The connections between all nodes in the list form the optimized path, as illustrated by the black solid line in the figure. As a result, the number of turning points in the path is reduced from 6 to 4, and unnecessary collinear nodes are eliminated. The method proposed in this paper retains only the necessary turning points in the path, achieving effective optimization.

### 3.4. Local Smoothing Using Cubic Spline Curves

At polyline turns, mobile robots may experience mechanical limitations, leading to stalling, and sharp changes in slope along the path may cause mechanical wear during turns. Therefore, the path needs to be smoothed to form a continuous and smooth path.

**Figure 6:**
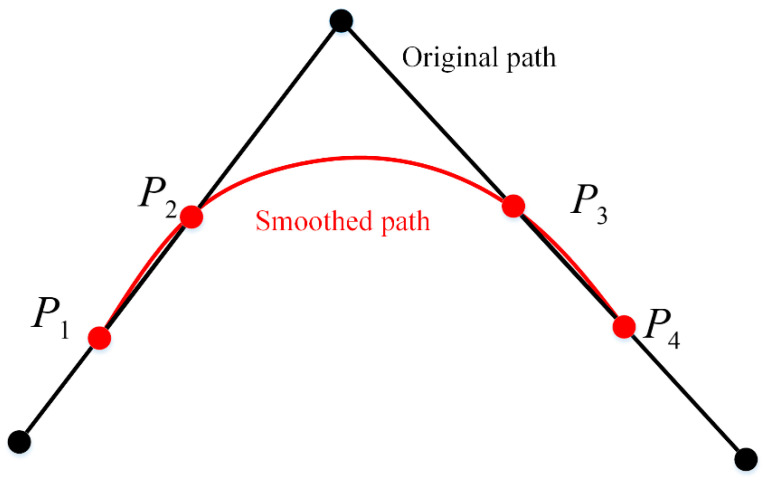
Illustration of Path Smoothing.

Many studies smooth the entire path using interpolation. However, in maps with densely distributed obstacles, the smoothed path may intersect with obstacles. This paper retains the straight sections of the path and only applies cubic spline interpolation at the turning points for optimization. As shown in Figure 6, the four nodes $P_{1},P_{2},P_{3},P_{4}$ before and after the turning point are selected as control points for cubic spline interpolation. The turning point itself is not used to avoid collisions between the smoothed path and obstacles. The second segment $\left[P_{2},P_{3}\right]$ of the interpolated path smooths the turn into an arc, while the first segment $\left[P_{1},P_{2}\right]$ and third segment $\left[P_{3},P_{4}\right]$ ensure that the interpolated curve connects smoothly with the original straight path at the junctions.

In the three intervals between the control points $P_{1},P_{2},P_{3},P_{4}$, namely $\left[P_{1},P_{2}],[P_{2},P_{3}],[P_{3},P_{4}\right]$, cubic functions $S_{1}(x),S_{2}(x),S_{3}(x)$ are used for interpolation, as shown in Equations (8)–(10).

(8) $$S_{1}(x)=a_{1}+b_{1}(x-x_{P_{1}}))+c_{1}{\left(x-x_{P_{1}}\right)}^{2}+d_{1}{\left(x-x_{P_{1}}\right)}^{3}$$

(9) $$S_{2}(x)=a_{2}+b_{2}(x-x_{P_{2}}))+c_{2}{\left(x-x_{P_{2}}\right)}^{2}+d_{2}{\left(x-x_{P_{2}}\right)}^{3}$$

(10) $$S_{3}(x)=a_{3}+b_{3}(x-x_{P_{3}}))+c_{3}{\left(x-x_{P_{3}}\right)}^{2}+d_{3}{\left(x-x_{P_{3}}\right)}^{3}$$

**Figure 7:**
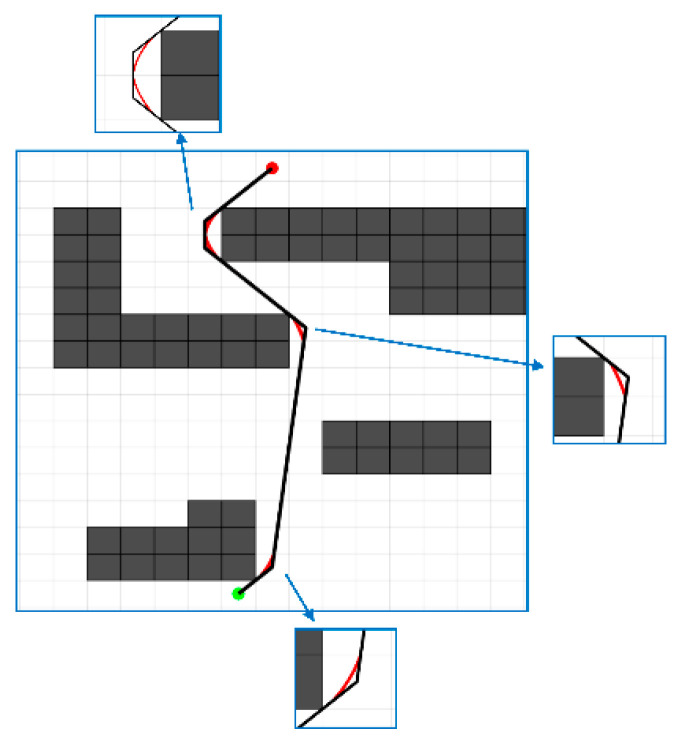
The result of local path smoothing.

Since the curve passes through each control point and the first and second derivatives of the segmented curves are equal at the two intermediate points, the coefficients can be solved based on the conditions in Equation (11), and the final interpolated curve can be plotted. The result is shown in Figure 7.

(11) $$\begin{array}{l} S_{i}(x_{p_{i}})=y_{p_{i}},i=1,2,3\\ S_{i}(x_{p_{i+1}})=S_{i+1}(x_{p_{i+1}}),i=1,2\\ {S^{\prime \prime }}_{i}(x_{p_{i+1}})={S^{\prime \prime }}_{i+1}(x_{p_{i+1}}),i=1,2\\ {S^{\prime \prime }}_{i}(x_{p_{i+1}})={S^{\prime \prime }}_{i+1}(x_{p_{i+1}}),i=1,2 \end{array}$$

## 4. Simulation Experiments and Result Analysis

The effectiveness and reliability of the VNS-BA* algorithm were verified through simulations conducted in the MATLAB 2018b environment. For comparison, this study reproduced an improved A* algorithm from [28] and an improved bidirectional A* algorithm from [29]. Since [29] did not explicitly specify the value of the weighting factor, the obstacle density factor proposed earlier was used as the weighting factor. Four maps of varying sizes and increasing complexity were designed, and the proposed algorithm was compared with the traditional A*, the improved A* algorithm [28], the bidirectional A* algorithm (BA*), and the improved bidirectional A* algorithm (improved BA*) [29]. In the maps, gray areas represent obstacle grids, red circles indicate the start points, green circles represent the goal points, yellow grids denote the forward path search from the start, blue grids denote the backward path search from the goal, black lines indicate the planned path, and red lines at path turns represent locally smoothed sections of the path planned by the proposed algorithm.

**Figure 8:**
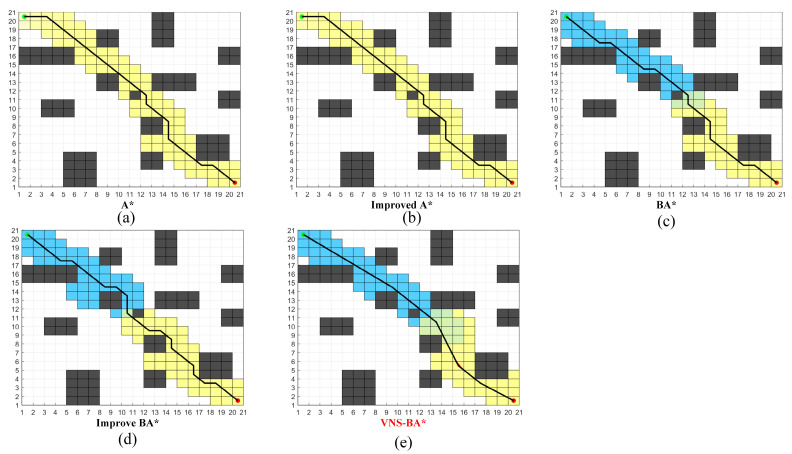
Performance of the five algorithms on Map A.

As shown in Figure 8, the paths generated by the five algorithms on a simple 20 × 20 map are presented. The specific data is shown in Table 2.

Due to the simplicity of the map structure, there were no significant differences in path length or the number of expanded nodes among the five methods. However, in terms of time efficiency, the three methods using the bidirectional search strategy performed significantly better than the two unidirectional algorithms. Furthermore, due to the use of the polyline optimization strategy, the proposed VNS-BA* algorithm produced paths with noticeably fewer turns and greater smoothness compared to the other four algorithms.

**Figure 9:**
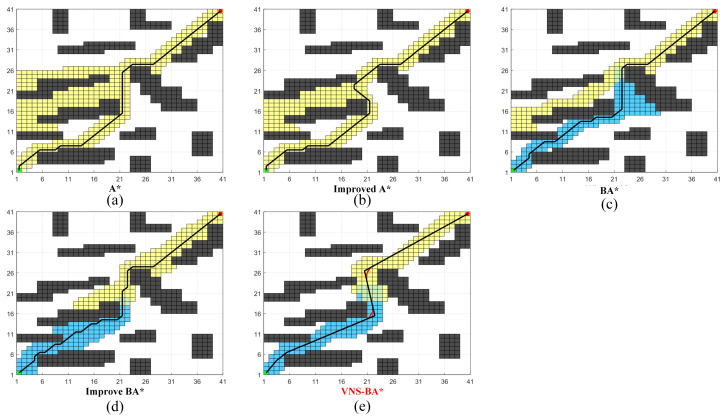
Performance of the five algorithms on Map B.

As the map complexity increases, zigzag-shaped obstacles are added in Map B, as shown in the Figure 9. The specific data is shown in Table 3.

Due to the presence of branching paths in the middle of the map, the traditional unidirectional A* algorithm and the improved A* [28] entered the wrong branch at the center of the map, leading to redundant searches and significantly longer planning times compared to the other three bidirectional algorithms. The BA* algorithm encountered an issue where the forward and backward paths did not converge in the middle of the map, resulting in numerous redundant search nodes. In the Improved BA* [29], the forward path also deviated from the backward search path, and the generated path contained many turns. The proposed VNS-BA* algorithm employs an improved bidirectional search strategy, adding a distance weight between the current node and the opposite path’s current node to the heuristic function. This modification allows the forward and backward paths to attract each other and meet in the middle, avoiding unnecessary searches and achieving optimized results across all five evaluated path quality metrics.

**Figure 10:**
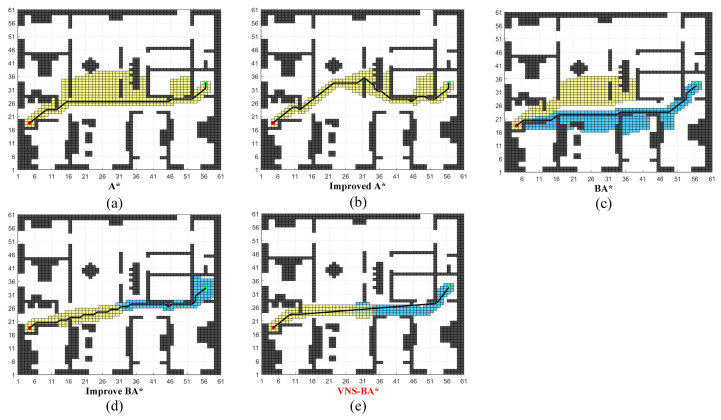
Performance of the five algorithms on Map C.

The 60 × 60 environment shown in Figure 10 simulates indoor scenarios such as home and office environments. The specific data is presented in Table 4.

The traditional A*, improved A* [28], and BA* algorithms experienced extended planning times due to many unnecessary searches. The improved A* generated paths that exhibited detours, failing to achieve global optimality. The Improved BA* [29], using the traditional 8-neighborhood search, produced paths with excessive turns, which were not favorable for robot navigation. In contrast, the proposed algorithm employs the 8-11-13 neighborhood search strategy, where, in the central part of the map, the line between the forward and backward paths is parallel to the *x*-axis with fewer obstacles, allowing nodes to expand using the 11-neighborhood. This reduces the number of node iterations and turns, thereby improving planning speed. Data analysis indicates that although the number of nodes explored by the proposed algorithm is not the lowest, the generated paths are optimized in terms of length, planning time, and smoothness.

**Figure 11:**
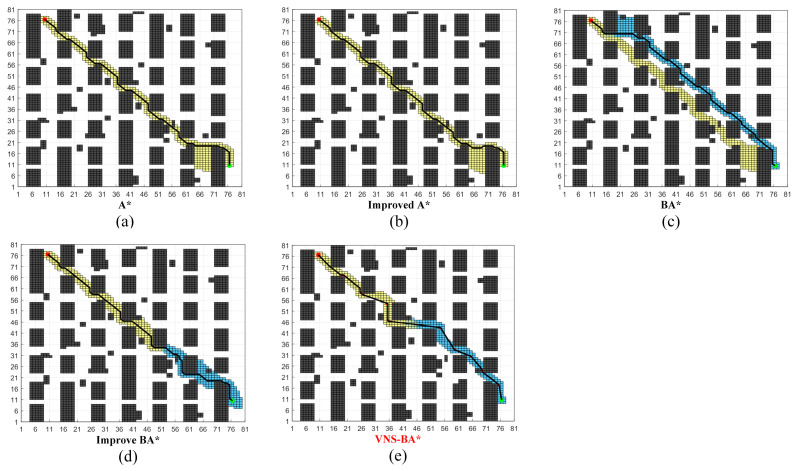
Performance of the five algorithms on Map D.

The 80 × 80 map shown in Figure 11 simulates environments such as ports and warehouses, where obstacles represent goods placed in the scene. The specific data is presented in Table 5.

As map complexity increased, all algorithms exhibited a significant rise in the number of turns and turn angles in their paths. However, VNS-BA* still demonstrated superior performance in terms of path smoothness and planning time, validating its effectiveness in large, complex environments.

Based on the results above, the optimization rate of VNS-BA* compared to the other four algorithms in the four scenarios was calculated, as shown in Table 6.

From the analysis of the data in Table 6, it can be concluded that VNS-BA* shows significant improvement compared to traditional A*, improved A* [28], BA*, and improved BA* [29]. Specifically, in terms of path smoothness and planning time, VNS-BA* achieved a turn-point optimization rate above 40% for all scenarios, with a maximum of 80.96%. The turn-angle optimization rate ranged from a minimum of 28.44% to a maximum of 83.53%. The planning time of VNS-BA* was notably better than that of the unidirectional algorithms, with some improvement over the bidirectional algorithms as well. In simple environments, all algorithms had a low number of node iterations, and due to the larger number of nodes expanded per iteration by the proposed algorithm, negative optimization was observed in node reduction. However, as the size of the map increased, the optimization effect also progressively improved.

**Figure 12:**
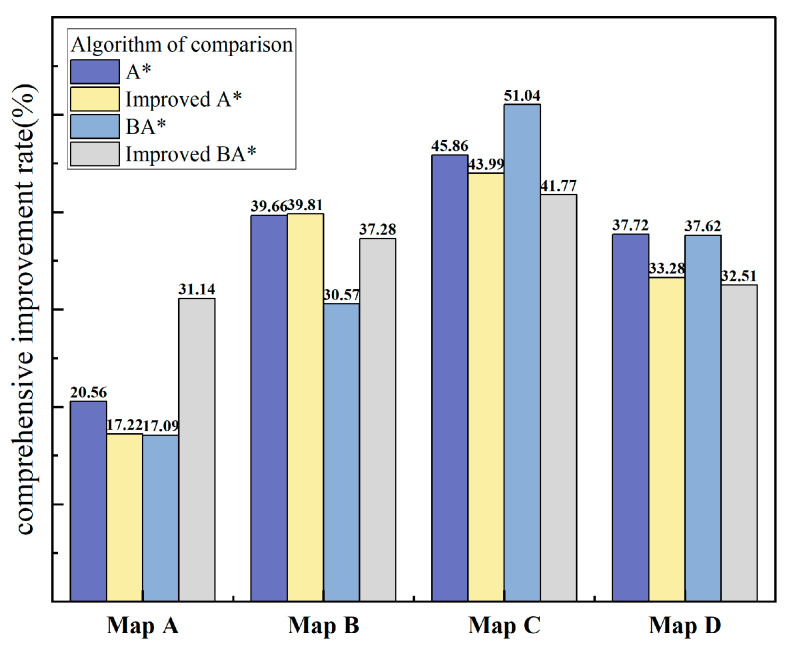
A grouped bar chart showing the overall optimization rate of VNS-BA* compared to the other four algorithms across different maps.

To comprehensively evaluate the optimization effect of the algorithm, the arithmetic mean method is used to calculate the overall optimization rate of the algorithm, and the results are displayed using a bar chart, as shown in Figure 12.

From the analysis of the combined bar charts, it can be concluded that the proposed VNS-BA* algorithm maintains a leading position even in simple environments, though the advantage is not pronounced. However, in relatively complex environments, such as map B, map C, and map D, the overall optimization rate of the paths generated by the proposed algorithm exceeded 30%, demonstrating a significant advantage. This confirms that VNS-BA* is effective across various scenarios.

## 5. Conclusions

To meet the demand for high-quality global path planning in different scenarios, this paper proposes the VNS-BA* algorithm, which efficiently generates smooth paths with low path costs. The algorithm introduces an improved node expansion method to selectively and efficiently expand nodes for pathfinding. Additionally, it enhances the search strategy by using the current node of the opposite path as the target in the bidirectional search, guiding the paths to converge at the midpoint of the map, and further improves the heuristic function to increase search speed. Finally, unnecessary turns are removed, and local smoothing is applied to remaining turns, resulting in a stable path suitable for robot navigation.

Simulation results in various scenarios demonstrate that, compared to traditional A*, BA*, and two other optimized algorithms, VNS-BA* effectively reduces the number of path turns and achieves favorable optimization in terms of planning time and the number of expanded nodes. However, the paths generated by the proposed algorithm are only applicable in static environments with known map information. When obstacle states change within the map, dynamic obstacle avoidance algorithms are still needed to mitigate collision risks, which will be the focus of future research.

## Figures and Tables

**Table 1:** Simulation results for different values of ω,η in the 4 test maps.

(ω,η)	Average Number of Nodes	Average Path Time (s)	Average Path Length
(0, 1)	97.5	1.6526	24.4121
(0.1, 0.9)	92.5	1.8305	25.1159
(0.2, 0.8)	91.75	1.8056	24.0503
(0.3, 0.7)	89	1.7324	24.1349
(0.4, 0.6)	79.75	1.2657	24.1159
(0.5, 0.5)	78	0.9382	24.2818
(0.6, 0.4)	83	1.2025	25.3870
(0.7, 0.3)	81.75	1.2419	24.3708
(0.8, 0.2)	83.75	1.2762	25.4154
(0.9, 0.1)	85.25	1.2658	25.3154
(1, 0)	85.5	1.3067	25.7689

**Table 2:** Comparison of Simulation Results of Different Algorithms in a 20 × 20 Map A.

Map	Map Size	Algorithms	Number of Nodes	Number of Turns	Turns Angles (Deg)	Path Time (s)	Path Length
A	20 × 20	A*	84	7	315	4.350	28.627
		Improved A* [28]	81	7	315	3.597	28.627
		BA*	88	10	450	2.155	28.627
		Improved BA* [29]	91	14	630	3.247	29.213
		VNS-BA*	100	4	208.967	2.553	27.476

**Table 3:** Comparison of Simulation Results of Different Algorithms in a 40 × 40 Map B.

Map	Map Size	Algorithms	Number of Nodes	Number of Turns	Turns Angles (Deg)	Path Time (s)	Path Length
B	40 × 40	A*	345	9	405	154.602	61.598
		Improved A* [28]	277	11	495	104.956	64.083
		BA*	286	12	540	28.129	61.598
		Improved BA* [29]	263	18	810	33.863	61.598
		VNS-BA*	252	4	289.823	20.419	61.263

**Table 4:** Comparison of Simulation Results of Different Algorithms in a 60 × 60 Map C.

Map	Map Size	Algorithms	Number of Nodes	Number of Turns	Turns Angles (Deg)	Path Time (s)	Path Length
C	60 × 60	A*	423	7	315	296.377	59.385
		Improved A* [28]	258	7	945	72.996	69.0833
		BA*	564	8	360	212.650	59.3848
		Improved BA* [29]	239	21	990	22.531	61.598
		VNS-BA*	257	4	163.072	12.330	57.517

**Table 5:** Comparison of Simulation Results of Different Algorithms in a 80 × 80 Map D.

Map	Map Size	Algorithms	Number of Nodes	Number of Turns	Turns Angles (Deg)	Path Time (s)	Path Length
D	80 × 80	A*	352	25	1125	108.308	103.297
		Improved A* [28]	344	27	1215	105.124	104.125
		BA*	611	24	1080	80.221	102.125
		Improved BA* [29]	392	30	1350	66.982	106.225
		VNS-BA*	355	14	672.95	35.345	103.700

**Table 6:** The improving ratio of VNS-BA* compared to other algorithms across different maps.

Algorithms of Comparison	Map	Map Size	Improving Ratio of Nodes	Improving Ratio of Turns	Improving Ratio of Angles	Improving Ratio of Time	Improving Ratio of Length
A*	A	20 × 20	−19.05%	42.86%	33.67%	41.31%	4.02%
	B	40 × 40	26.96%	55.56%	28.44%	86.80%	0.54%
	C	60 × 60	39.24%	42.86%	48.23%	95.84%	3.14%
	D	80 × 80	−0.85%	44.00%	39.91%	67.37%	−0.39%
Improved A* [28]	A	20 × 20	−23.46%	42.86%	33.67%	29.02%	4.02%
	B	40 × 40	9.02%	63.63%	41.45%	80.55%	4.40%
	C	60 × 60	0.39%	42.86%	82.74%	83.11%	10.83%
	D	80 × 80	3.20%	48.15%	44.61%	66.38%	4.08%
BA*	A	20 × 20	−13.64%	60%	53.56%	−18.47%	4.02%
	B	40 × 40	11.89%	66.67%	46.33%	27.40%	0.54%
	C	60 × 60	54.43%	50.00%	54.70%	94.20%	1.869%
	D	80 × 80	41.90%	41.67%	50.15%	55.94%	−1.54%
Improved BA* [29]	A	20 × 20	−9.89%	71.43%	66.83%	21.37%	5.98%
	B	40 × 40	4.18%	77.78%	64.22%	39.70%	0.54%
	C	60 × 60	−7.53%	80.96%	83.53%	45.28%	6.63%
	D	80 × 80	9.44%	53.33%	50.15%	47.23%	2.38%

## Data Availability

No new data were created or analyzed in this study. Data sharing is not applicable to this article.
